# Effects of pro‐inflammatory cytokines on cannabinoid CB_1_ and CB_2_ receptors in immune cells

**DOI:** 10.1111/apha.12474

**Published:** 2015-03-10

**Authors:** L. Jean‐Gilles, M. Braitch, M. L. Latif, J. Aram, A. J. Fahey, L. J. Edwards, R. A. Robins, R. Tanasescu, P. J. Tighe, B. Gran, L. C. Showe, S. P. Alexander, V. Chapman, D. A. Kendall, C. S. Constantinescu

**Affiliations:** ^1^ Division of Clinical Neuroscience School of Medicine University of Nottingham Nottingham UK; ^2^ Division of Neuroscience School of Life Sciences University of Nottingham Nottingham UK; ^3^ Division of Immunity School of Life Sciences University of Nottingham Nottingham UK; ^4^ Department of Neurology Colentina Hospital University of Medicine and Pharmacy Carol Davila Bucharest Romania; ^5^ The Wistar Institute Philadelphia PA USA

**Keywords:** cannabinoid receptor 1, cannabinoid receptor 2, inflammatory cytokines, multiple sclerosis, regulation

## Abstract

**Aims:**

To investigate the regulation of cannabinoid receptors CB_1_ and CB_2_ on immune cells by pro‐inflammatory cytokines and its potential relevance to the inflammatory neurological disease, multiple sclerosis (MS).

CB_1_ and CB_2_ signalling may be anti‐inflammatory and neuroprotective in neuroinflammatory diseases. Cannabinoids can suppress inflammatory cytokines but the effects of these cytokines on CB_1_ and CB_2_ expression and function are unknown.

**Methods:**

Immune cells from peripheral blood were obtained from healthy volunteers and patients with MS. Expression of CB_1_ and CB_2_ mRNA in whole blood cells, peripheral blood mononuclear cells (PBMC) and T cells was determined by quantitative real‐time polymerase chain reaction (qRT‐PCR). Expression of CB_1_ and CB_2_ protein was determined by flow cytometry. CB_1_ and CB_2_ signalling in PBMC was determined by Western blotting for Erk1/2.

**Results:**

Pro‐inflammatory cytokines IL‐1*β*, IL‐6 and TNF‐*α* (the latter likely NF‐*κ*B dependently) can upregulate CB_1_ and CB_2_ on human whole blood and peripheral blood mononuclear cells (PBMC). We also demonstrate upregulation of CB_1_ and CB_2_ and increased IL‐1*β*, IL‐6 and TNF‐*α* mRNA in blood of patients with MS compared with controls.

**Conclusion:**

The levels of CB_1_ and CB_2_ can be upregulated by inflammatory cytokines, which can explain their increase in inflammatory conditions including MS.

Activation of cannabinoid receptors, CB_1_ and CB_2_ by endocannabinoids or exogenous ligands can suppress inflammation (Tanasescu & Constantinescu 2010). The endocannabinoid (EC) system consists of the G‐protein‐coupled receptors CB_1_ and CB_2_, the endogenous ligands such as anandamide and 2‐arachidonylglycerol and the synthesizing and metabolizing enzymes (Alexander & Kendall 2007). Exogenous cannabinoid compounds such as phytocannabinoids Δ^9^ tetrahydrocannabinol and cannabidiol have immunomodulatory properties (Tanasescu & Constantinescu 2010). They inhibit pro‐inflammatory cytokines (Klein *et al*. 2000); (Jean‐Gilles *et al*. 2010), suppress T‐cell proliferation (Katona *et al*. 2005), induce T‐cell apoptosis (Rieder *et al*. 2010) and reduce migration and adhesion of immune cells. CB receptors are expressed on immune cells including B cells, T cells, neutrophils and NK cells, with CB_2_ levels known to be higher than those of CB_1_ (Galiegue *et al*. 1995). Their widespread location in the immune system allows CB receptor activation to regulate cytokine levels.

Both CB receptors are also expressed in CNS cells such as neurones, oligodendrocytes, astrocytes and microglia (Molina‐Holgado *et al*. 2002, Sheng *et al*. 2005, Stella 2009, Pertwee 2010). All these cells can secrete inflammatory mediators that are implicated in the pathogenesis of neuro‐inflammatory and neurodegenerative diseases such as multiple sclerosis (MS) (Hemmer *et al*. 2006). MS is a chronic inflammatory disease of the CNS white and grey matter which is characterized by demyelination, axonal injury and gliosis (Compston & Coles 2008). Inflammatory cytokines such as tumour necrosis factor (TNF)‐*α*, interleukin (IL)‐1*β* and IL‐6 are elevated in lesions, cerebrospinal fluid and blood of patients with MS (Kahl *et al*. 2002, Filion *et al*. 2003, Kleine *et al*. 2003, Malamud *et al*. 2003). These cytokines are thought to play a role both in the CNS and in the periphery in neuro‐inflammatory disease including MS (Compston & Coles 2008).

Neuroprotective and immunomodulatory effects of cannabinoids have been demonstrated in the animal model of MS, experimental autoimmune encephalomyelitis (EAE) (Pryce *et al*. 2003) (Arevalo‐Martin *et al*. 2003). Beneficial effects were associated with diminished CNS mRNA expression of pro‐inflammatory cytokines (Croxford & Miller 2003). Cannabinoids have also shown symptomatic benefit in human MS clinical trials (Pertwee 2002, Zajicek *et al*. 2003, Kavia *et al*. 2010, Rog 2010) where reduced pain, sleep disturbance, bladder overactivity, tremor and spasticity have been observed.

Although CB signalling and the regulation of cytokines by cannabinoids are well documented (De Jesus *et al*. 2006, Rubio‐Araiz *et al*. 2008), only a few recent studies, in rodents mostly, have looked at the effects of inflammatory cytokines on the cannabinoid system. An increase in interferon (IFN)‐*γ* modulated microglial CB_2_ receptors in neuropathic pain and MS mouse models (Maresz *et al*. 2005, Racz *et al*. 2008). Also, CB_2_ upregulation in chronic EAE correlated highly with the production of pro‐inflammatory cytokines (Loría *et al*. 2008). CB_1_ receptors were also upregulated by the Th2 cytokine IL‐4 (which downregulates both Th1 and Th17 cytokines involved in the pathogenesis of MS and EAE) and by cannabinoids themselves in human T lymphocytes (Börner *et al*. 2007, Borner *et al*. 2008) while IFN‐*γ* was not shown to have any effect on CB_1_ expression by human T cells (Börner *et al*. 2007). In other studies in EAE, CB_2_ was shown to be upregulated in a progressive manner on specific post‐immunization days in the presence of cytokines IFN‐*γ*, IL‐17, IL‐4, IL‐10, IL‐1*β*, IL‐6 and TNF‐*α* (Lou *et al*. 2011), while CB_1_ receptor expression was downregulated on same days (Lou *et al*. 2012). Those studies suggest that manipulation of CB and CB receptors may have therapeutic value in MS.

In this study, we investigate the effects of the above pro‐inflammatory cytokines on the expression of CB_1_ and CB_2_ on immune cells from both healthy subjects and patients with MS. We also show an upregulation of mRNA for CB receptors and for TNF‐*α*, IL‐1 and IL‐6 in peripheral blood of people with MS compared to healthy controls.

## Materials and methods

### Donors and RNA extraction from blood

The study conforms with guidelines for *Acta Physiologica* (Persson 2013). Whole blood was obtained from 20 patients with MS (relapsing remitting, *n* = 12; secondary progressive, *n* = 6; and primary progressive, *n* = 2). These included 13 females and 7 males aged 28–64 years; mean Expanded Disability Status Scale (EDSS) score (Kurtzke 1983) = 4.6; range, 2.0–7.5. The patients were free of MS‐specific immunomodulatory, immunosuppressive or monoclonal antibody therapies for at least 2 months.

Nineteen age‐ and gender‐matched healthy volunteer subjects (14 females and 5 males, aged 27–62 years), not taking medicines, were used as controls. Peripheral blood mononuclear cells (PBMC) were isolated from heparinized whole blood obtained from healthy adult donors (8 females, 8 males; age 21–56 years) by density gradient centrifugation. Cells were resuspended in 10% FCS/RPMI, transferred to 24‐well plates at 1 × 10^6^/well and unstimulated or stimulated with IL‐6 (100 ng mL^−1^), IL‐1*β* (100 ng mL^−1^) or TNF‐*α* (25 ng mL^−1^) (Peprotech, London, UK) in the absence or the presence of the NF‐*κ*B inhibitor SN50 (18 *μ* m) (Calbiochem, Nottingham, UK) and incubated at 37 °C (5% CO_2_) for 18 h.

In a separate experiment comparing MS and control PBMC responses to pro‐inflammatory cytokines, PBMC were isolated as above from 12 patients with MS different from the above (10 females, 2 males; mean ± SD age 48.3 ± 13.8 years) and 12 healthy controls (7 females, 5 males, males; mean ± SD age 42.8 ± 10.2 years). Five were not receiving and 7 were receiving disease‐modifying treatment (5 interferon, 2 glatiramer acetate). Frozen PBMC were thawed and unstimulated or stimulated with IL‐6, IL‐1*β* or TNF‐*α* (100 pg mL^−1^). Expression of CB_1_ and CB_2_ mRNA and protein was analysed by qRT‐PCR and flow cytometry respectively.

### Generation of polyclonally activated T cells (T‐cell blasts)

Phytohemagglutinin (PHA)/IL‐2‐stimulated T cells were obtained from PBMC as shown before (Fahey *et al*. 2007) and after 24‐h serum starvation stimulated with IL‐6, IL‐1*β* (100 ng mL^−1^) or TNF‐*α* (25 ng mL^−1^) for a further 18 h.

### RNA extraction and quantification

This was performed as previously described (Fahey *et al*. 2007, Jean‐Gilles *et al*. 2009).

### Whole blood preparation for flow cytometry

0.5 mL of heparinized venous blood was unstimulated or stimulated with IL‐1*β*, IL‐6 (100 ng mL^−1^) or TNF‐*α* (25 ng mL^−1^) and incubated for 18 h (37 °C; 5%CO_2_). 2 mmol L^−1^ EDTA (pH 8) was added to tubes and incubated for 15 min (RT). Aliquotes were exposed to FACS lysing and FACS permeabilizing solution (BD Biosciences, Oxford, UK) and stained with antibodies CB_1_ [1 : 50] (PA1‐745), CB_2_ [1 : 50] (PA1‐744) (Affinity Bioreagents, Golden, CO, USA) or control normal rabbit IgG [1 : 40] (ProSci, Poway, CA, USA), then incubated with PE‐conjugated goat anti‐rabbit IgG [1 : 10] (Invitrogen, Paisley, UK) and were fixed in 1% formaldehyde/PBS for flow cytometry (Beckman‐Coulter Altra Sorter, High Wycombe, UK).

### Reverse‐transcriptase reaction and quantitative real‐time polymerase chain reaction

These studies were performed as previously described (Fahey *et al*. 2007) on a Mx4000 or Mx3005 platform (Stratagene, La Jolla, CA, USA). Reaction conditions are available on request. Primer sequences are shown in Table 1. Beta‐2‐microglobulin (B2MG) (PBMC, T cells) and large human ribosomal protein‐PO (RPLPO) (whole blood) were used as housekeeping genes for normalization of data.

**Table 1:** Primers used for PCR

B2MG for	5′‐CTC CGT GGC CTT AGC TGT G‐3′
B2MG rev	5′‐TTT GGA GTA CGC TGG ATA GCC T‐3′
RPLPO for	5′‐CCA CGC TGC TGA ACA TGC T‐3′
RPLPO rev	5′‐TCG AAC ACC TGC TGG ATG AC‐3′
CB_1_ for	5′‐CTG GAA CTG CGA GAA ACT G‐3′
CB_1_ rev	5′‐CGC ATA CAC GAT GAA CAG AAG‐3′
CB_2_ for	5′‐GCC TCT TCC CAA TTT AAA CAA C‐3′
CB_2_ rev	5′‐AGT CAG TCC CAA CAC TCA TC‐3′
IL‐6 for	5′‐TCA ATG AGG AGA CTT GCC TG‐3′
IL‐6 rev	5′‐GAT GAG TTG TCA TGT CCT GC‐3′
IL‐1*β* for	5′‐GGA TAT GGA GCA ACA AGT GG‐3′
IL‐1*β* rev	5′‐ATG TAC CAG TTG GGG AAC TG‐3′
TNF‐*α* for	5′‐ACA AGC CTG TAG CCC ATG TT‐3′
TNF‐*α* rev	5′‐AAA GTA GAC CTG CCC AGA CT‐3′

for, forward; rev, reverse.

mRNA results were based on normalized ratio to the internal standard expression (RPLPO for blood or B2MG for PBMC and T cells), and significance (*P *≤* *0.05) in differences between the groups was determined by *t*‐test and one‐way repeated measures anova.

### Intracellular staining and flow cytometry

Peripheral blood mononuclear cells thawed from 12 patients with MS and 12 healthy controls were unstimulated or stimulated with cytokines then incubated for 24 h. For some experiments (not shown), conjugated anti‐CD4, CD8 (T cells), CD3‐, ‐CD14, ‐CD16 and ‐CD19 (PBMC) mouse monoclonal antibodies were added. After fixation and permeabilization, they were stained with CB1‐APC (and isotype control) and CB2 primary antibody (R&D), incubated for 30 min and then stained with FITC secondary antibody and isotype control (R&D). Data were analysed using weasel v3.1.

For T‐cell experiments, the same above reagents as for whole blood were used. CB_1_ and CB_2_ protein abundance was using winmdi software. Changes in mean fluorescence intensity [geometric (g) mean] and/or % of positive cells were calculated by comparing negative control (no anti‐CB_1_ or CB_2_ antibodies used), unstimulated and stimulated (with cytokines) cells. Positive cells were gated in two‐parameter histograms and/or shifts (increase in mean or median fluorescence intensity (MFI) were gated within marker M1 for single‐parameter histograms and averaged across whole population.

### Western blotting

Peripheral blood mononuclear cells were in 2%FCS/RPMI with or without cytokines, CB1/CB2 agonist HU‐210 (Tocris, Bristol, UK) [100 nm] in the presence or absence of CB_1_ antagonist SR141716A (SR1, rimonabant) and CB_2_ antagonist SR144528 (SR2) [300 nm] (gifts from Sanofi, Guildford, UK) and were centrifuged for 10 min (300 ***g***, 4 °C). The supernatant was discarded and lysis buffer [with 20 mmol L^−1^ Tris, 1 mmol L^−1^ EGTA and protease inhibitor (P8340; Sigma, Gillingham, UK)] was added. Samples were homogenized for 30 min (4 °C) and supernatant extracted. To quantify protein, Lowry test was conducted for homogenates and human recombinant CB_1_ and CB_2_ proteins (Axxora, UK) used as positive controls, and protein was equally loaded onto gels. 2× solubilization buffer [containing Tris (20 mmol L^−1^) (Invitrogen), glycerol, sodium dodecyl sulphate (10%) (SDS), *β*‐mercaptoethanol and bromophenol blue (Sigma)] was added [1 : 2], and samples were heated to 100 °C for 5 min. Proteins (20 *μ*g) were loaded onto 10% SDS gels and resolved by SDS‐polyacrylamide gel electrophoresis with a standard marker ladder of known molecular weights run alongside (45 min, 200 V). Following transfer onto nitrocellulose membranes (Amersham Biosciences, Little Chalfont, UK) (60 min, 100 V, 4 °C) and staining with Ponceau S (Sigma), blots were blocked in 1 g/20 mL non‐fat dry milk in 0.1% Tris‐buffered saline–Tween 20 (pH 7.6) for 60 min. Separated membranes were incubated overnight (4 °C) with anti‐CB_1_ (1 : 500), anti‐CB_2_ (1 : 1000) (Affinity Bioreagents), total or phosphorylated (p)‐mitogen‐activated protein kinase (MAPK) 42/44 (Erk1/2) (1 : 1000) antisera (Cell Signalling Biotechnology, Hitchin, UK). Blots were then washed and incubated with secondary antibody horseradish peroxidase‐conjugated goat anti‐rabbit IgG (Amersham Biosciences) in blocking buffer (1 : 2000) for 60 min (RT) and reprobed with *β*‐actin (1 : 4000) antibody (Abcam, Cambridge, UK) as a loading control using enhanced chemoluminescent reagents, hyperfilm (Amersham) and Kodak reagents for development. Specific immunoreactive bands were analysed using a Bio‐Rad GS‐710 imaging scanning densitometer. Protein differences between samples were analysed based on optical density (OD) of each band and by two‐tailed *t*‐test with *P *≤* *0.05. Differences were explored using one‐way repeated measurements anova.

## Results

### CB_1_ and CB_2_ expression in cytokine‐stimulated whole blood, PBMC and PHA/IL‐2 induced T cells of normal subjects

**Figure 1:**
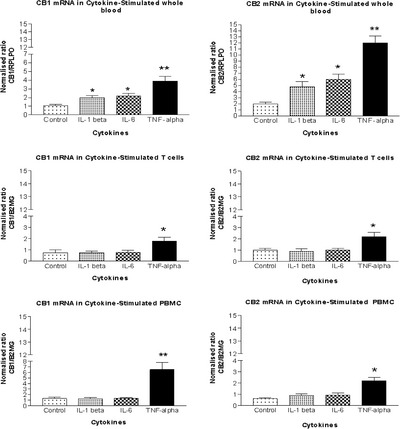
CB1 and CB2 mRNA expression in whole blood, T cells and PBMC. Cytokine stimulation was with IL‐6 [100 ng mL^−1^], IL‐1*β* [100 ng mL^−1^] and TNF‐*α* [25 ng mL^−1^] mRNA levels for CB1 (left) and CB2 (right) (arbitrary units) are presented as mean ± SEM normalized to RPLPO in whole blood and to B2MG in T cells and PBMC. *t‐*test for independent samples: * = *P *<* *0.05; **= *P *<* *0.025; *n* = 4–5 experiments per condition.

The effects of IL‐6, IL‐1*β* and TNF‐*α* on CB_1_ and CB_2_ receptor gene expression in whole blood, PBMC and T cells are shown in Figure 1. TNF‐*α* significantly induced CB_1_ and CB_2_ mRNA levels in whole blood (*P *=* *0.02, *P *=* *0.01), T cells (*P *=* *0.04, *P *=* *0.04) and PBMC (*P *=* *0.01, *P *=* *0.04) compared to unstimulated cells. In each cell population, the expression of CB receptor mRNA levels induced by TNF‐*α* was 2–3 times higher than levels induced by IL‐6 and IL‐1*β*. Although IL‐1*β* and IL‐6 significantly increased CB_1_ (*P *=* *0.04, *P *=* *0.04) and CB_2_ (*P *=* *0.03, *P *=* *0.02) mRNA levels in whole blood, induction by these cytokines in T cells did not reach significance. In PBMC, a non‐significant increase was noted for CB_2_ (*P *=* *0.06 for IL‐6; *P *=* *0.07 for IL‐1*β*) and for CB_1_ (*P *=* *0.12 for IL‐6; *P *=* *0.19 for IL‐1*β*). These differences may reflect a direct or indirect contribution of CB mRNA induction in polymorphonuclear leucocytes.

**Figure 2:**
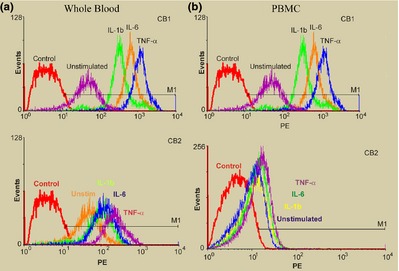
(a–b) CB1 and CB2 protein expression determined by flow cytometry. Changes were measured as increase in mean fluorescence intensity (MFI) of gated lymphocytes/monocytes (a) CB1 and CB2 in whole blood (left panel). (b) CB1 and CB2 in PBMC (right panel). See, text for significance levels. Results shown are representative of five independent experiments. Control = rabbit IgG.

The effects of IL‐6, IL‐1*β* and TNF‐*α* on the CB_1_ and CB_2_ receptor at the protein level assessed using flow cytometry are shown in Figure 2. Although all three pro‐inflammatory cytokines increased CB_1_ and CB_2_ protein expression levels, stimulation with TNF‐*α* induced higher levels than IL‐6 or IL‐1*β* in whole blood and PBMC (Fig. 2a,b). CB_1_ and CB_2_ immunopositivity in whole blood gated for monocytes/lymphocytes was confirmed to be increased by TNF‐*α* (CB_1_ MFI = 973 ± 243, *P *=* *0.001 and CB_2_ MFI = 206 ± 56, *P *=* *0.02) when compared to unstimulated samples (MFI = 42.1 ± 14.1). CB_1_ protein MFI was also increased in whole blood stimulated with IL‐1*β* (317.4 ± 64.5, *P *=* *0.009) and IL‐6 (594.6 ± 130.8, *P *=* *0.005). CB_2_ protein MFI induced by IL‐1*β* showed a non‐significant increase (81.5 ± 20.4, *P *=* *0.06), and with IL‐6, it was significantly increased (106.3 ± 28.7, *P *=* *0.05) (Fig. 2a). PBMC stimulated with cytokines showed increases in CB_1_ (MFI: TNF‐*α *= 84.9 ± 17.8; *P *=* *0.02); IL‐6 (49.1 ± 1.1; *P *=* *0.04); IL‐1*β* (32.1 ± 7.0; *P *=* *0.04). CB_2_ protein levels (MFI for TNF‐*α *= 13.5 ± 4.0; *P *=* *0.08; IL‐6: 11.9 ± 3.0; *P *=* *0.09; IL‐1*β*: 10.5 ± 2.9; *P *=* *0.12) only showed an insignificant increase compared to unstimulated (MFI = 9.0 ± 2.7) samples (Fig. 2b). CB receptor protein expression on T cells showed similar regulation by cytokines to that of PBMC (data not shown).

### MAPK (Erk1/2) activation in HU‐210‐stimulated PBMC

**Figure 3:**
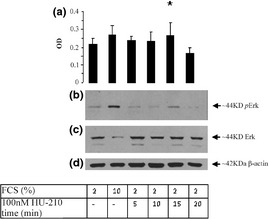
(a) Densitometry measurements in phosphorylated (*p)* ERK response to stimulation. Response of PBMCs to FCS 2 and 10%, followed by a time‐dependent (5–20 min) response to 100 nm HU‐210. The 2 vs. 10% FCS on these blots demonstrates a well‐established upregulation of *p*ERK by high concentrations of FCS. The difference between the average OD of *p*ERK at 15‐min stimulation was statistically significant compared with 2% FCS (*P *= 0.03, *n* = 3 experiments, indicated with asterisk). The highest level of *p*ERK with exposure to 100 nm HU‐210 was observed after 15 min (average OD = 0.25 ± 0.08) with a 23% increase (b) Phosphorylated ERK 42/44 (*p*ERK, 42 kDa) and (c) Total Erk 42/44 (Erk) (d) Beta‐actin was used as housekeeping protein; levels remained unchanged.

To confirm that CB receptors were functional in PBMC, HU‐210 (100 nm), a non‐selective CB_1_ and CB_2_ receptor agonist, was used to activate them. HU‐210 was chosen due to its potency in preference to weaker selective agonists (Alexander & Kendall 2007). HU‐210‐stimulated PBMC showed a transient enhancement of pMAPK42/44 levels (23% increase; *P *=* *0.03) compared to control (FCS 2%; OD_avg_ = 0.22 ± 0.04) to peak at 15 min (Fig. 3) although no significant increase in total MAPK was observed. Immunoreactivity of p42 was more prominent than p44. Beta‐actin immunoreactivity was unchanged.

### MAPK (Erk1/2) activation in cytokine‐stimulated PBMC

**Figure 4:**
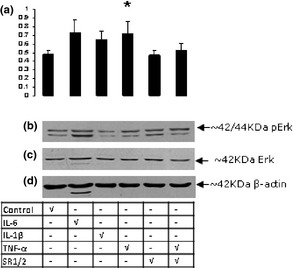
Immunoreactivity for *p*Erk 42/44 and effects of cytokine treatment in PBMC. (a) Densitometry measurements from three experiments. TNF‐*α*‐stimulated cells express significantly higher *p*Erk levels than unstimulated cells or TNF‐*α*‐stimulated cells in the presence of SR1 and 2 NF‐kB inhibitors, as indicated by asterisk. At 42 kDa, TNF‐*α* induced an increase in *p*Erk levels (OD avg = 0.72 ± 0.14; *P *= 0.03) compared to unstimulated samples, and its effects were reduced synergistically by SR1 and SR2 (OD avg = 0.53 ± 0.08; 26%; *P *= 0.04). *t‐*test for independent samples with *P *< 0.05; *n* = 3. (b) Bands corresponding to *p*Erk 42/44 were detected while (c) only bands at 42 kDa were detected for total Erk (ErK); this showed no significant change. (d) *β*‐actin showed no significant change.

Total and phosphorylated (p)MAPK42/44 immunoreactivity were also assessed in cytokine‐stimulated PBMC, with or without exposure to the cannabinoid receptor antagonists SR 141716 and SR 144528 (SR1 and SR2), by reprobing of blots (Fig. 4). Our preliminary work showed that the combination of SR1 and SR2 rather than individual antagonist use is typically needed to antagonize HU‐210 effectively in immune cells. No significant change was observed in total MAPK42/44 immunoreactivity (OD_avg_ = 0.20 ± 0.03) whereas pMAPK42/44 was increased in the presence of cytokines, potentially reflecting CB receptor response to cytokine stimulation in PBMC. These levels were decreased close to basal levels (OD_avg_ = 0.21 ± 0.05) in the presence of SR1 and SR2 (Fig. 4). TNF‐*α* induced the highest level of pMAPK42/44 immunoreactivity (OD_avg_ = 0.72 ± 0.14; *P *=* *0.03); SR1/SR2 reduced it (OD_avg_ = 0.53 ± 0.08; *P *=* *0.04) while remaining above basal levels. Beta‐actin immunoreactivity remained unchanged.

### CB_1_ and CB_2_ immunoreactivity and functional response in cytokine‐stimulated PBMC

**Figure 5:**
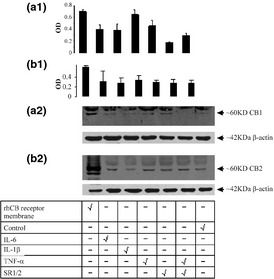
(a) a1. Densitometry graph of the Western blots (*n* = 3) for CB1 at 60 kDa. a2. Western blots showing immunoreactivity to CB1 antibody. Bands were detected at 32 (not shown) and 60 kDa corresponding to CB1. Lower blot (a2) is the corresponding *β*‐actin. (b) b1. Densitometry graph of the Western blots (*n* = 3) for CB2. b2 shows 60 kDa band for CB2 and below; lower blot (b) is its corresponding *β*‐actin. TNF‐*α* increased CB2 levels (OD avg = 0.34 ± 0.07; 17% increase), which was reduced in the presence of receptor antagonists (OD avg = 0.27 ± 0.07). TNF‐*α* increased CB1 (OD avg = 0.65 ± 0.13; 55% increase; *P *< 0.025). No changes in corresponding *β*‐actin were observed.

CB_1_ and CB_2_ immunoreactivity was assessed by Western blot in cytokine‐stimulated PBMC with or without the cannabinoid receptor antagonists SR141716A (SR1) and SR144528 (SR2) (300 nm each). Two bands were detected at around 60 and 37 kDa possibly corresponding to CB_1_ dimer and monomer, respectively, and a band migrating at approx. 60 kDa representing CB_2_ monomer (Fig. 5). These band sizes were similar to those obtained in the previous literature (Nunez *et al*. 2004, De Jesus *et al*. 2006) and to those of reference human CB1 recombinant protein (Fig. 5, left lane). CB_1_ and CB_2_ protein expression was upregulated by exposure to cytokines and downregulated in the presence of SR1 and SR2. The maximum OD increase was 55% (*P *=* *0.02) for CB_1_ and 17% for CB_2_ (*P *=* *0.07), possibly because of a higher baseline expression of CB_2_. No significant changes were observed in beta‐actin immunoreactivity.

### Effect of NF‐kB inhibitor treatment on cannabinoid receptor and TNF‐α gene expression in PBMC

**Figure 6:**
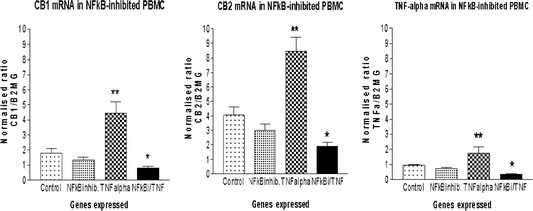
Effects of NF‐*κ*B inhibitor SN50 [18 *μ* m] on CB1(left), CB2 (middle) and TNF‐*α* (right) mRNA in unstimulated and TNF‐*α*‐ stimulated PBMC. The NF‐*κ*B inhibitor (SN50) decreased expression levels of each gene by more than a half in TNF‐*α*‐stimulated PBMC. Data represent mean average ±SEM; *n* = 4. *t*‐test *= *P *< 0.05; **P < 0.005.

NF‐*κ*B is a key transcription factor for several inflammatory mediators including TNF‐*α*. Given that this cytokine induced the highest levels of CB_1_ and CB_2_ expression (see above), we investigated its influence on cannabinoid receptor and TNF‐*α* gene expression in PBMC unstimulated or stimulated with TNF‐*α*, in the presence or absence of the NF‐*κ*B inhibitor SN50 (18 *μ* m). SN50 significantly decreased the levels of CB_1_, CB_2_ and TNF‐*α* mRNA (*P *=* *0.004, *P *=* *0.002 and *P *=* *0.004, respectively) compared to those of unstimulated cells and also decreased their expression in TNF‐*α*‐stimulated PBMC (all *P *<* *0.05) (Fig. 6). These results indicate that TNF‐*α* induction of CB_1_, CB_2_ and TNF‐*α* itself involves the NF‐*κ*B signalling pathways.

### Cytokine, CB_1_ and CB_2_ mRNA expression in blood from MS patients

**Figure 7:**
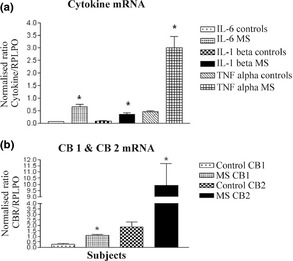
Cytokine and cannabinoid receptor mRNA results were based on normalized ratio of mean mRNA to mean RPLPO gene. (a) TNF‐*α*, IL‐6, IL‐1*β* and cannabinoid receptors (b) CB_1_ and CB_2_ mRNA expression measured by qPCR in control (*n* = 19) vs. MS (*n* = 20) blood. Data are displayed as mean ± SEM of 3–4 experiments. Asterisks (*) = *P* < 0.025 unpaired Student's *t*‐test.

mRNA levels for IL‐6, IL‐1*β* and TNF‐*α* were significantly increased in patient with MS blood compared to that of controls. IL‐6 showed the largest increase (17‐fold; *P *=* *0.01) in the MS group while IL‐1*β* showed fivefold (*P *=* *0.02) and TNF‐*α* showed sixfold (*P *=* *0.01) increase (Fig. 7a). CB_1_ and CB_2_ mRNA were also significantly elevated in MS blood compared to that of controls. CB_1_ and CB_2_ gene expression was increased approximately four‐ (*P *=* *0.02) and fivefold (*P *=* *0.01), respectively, in patients with MS (Fig. 7b).

### CB_1_ and CB_2_ mRNA and protein expression in MS vs. healthy control cytokine‐stimulated PBMC

To compare inducibility of CB receptors by cytokines in normal human immune cells and those of patients with MS, we investigated the effects of IL‐1*β*, IL‐6 and TNF‐*α* on CB mRNA of PBMC from 12 normal control subjects and 12 age‐ and sex‐matched patients with MS. We used low concentration ranges (100 pg mL^−1^) that are close to those found in body fluids in inflammatory circumstances. In this sample, the same trends as with the higher concentrations used above in normal controls were noted (data not shown). Statistical significance and a trend is seen for CB_1_ and CB_2_ increase at baseline in patients with MS (*P *=* *0.04 and 0.07 respectively). IL‐6 increased both CB_1_ and CB_2_ mRNA in healthy controls (*P *=* *0.018 and 0.013 respectively). The results partially confirm those using higher cytokine concentrations, but indicate that the lower concentrations are less potent. The data suggest that MS patients’ PBMC tend to respond similarly to those of healthy subjects in terms of CB induction, however, starting from higher baseline expression levels. No significant differences were noted between untreated patients and those on immunomodulatory treatment.

**Figure 8:**
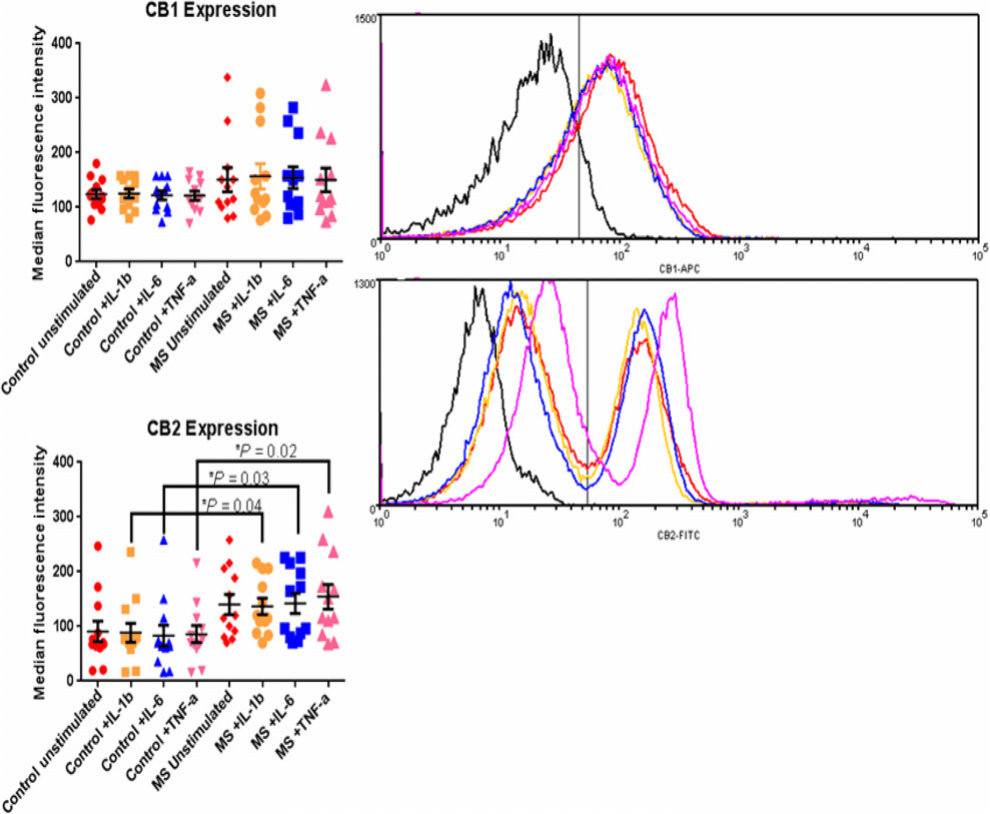
CB1 and CB2 expression, represented as median fluorescence intensity (left panel) in PBMC from 12 healthy controls and 12 MS patients under the specified stimulation conditions. Data are shown for lymphocyte/monocyte‐gated cells. The right panel is a representative histogram of CB_1_ and CB_2_ expression in an MS patient's PBMCs in different stimulation conditions. Colour legend, Black: Isotype Control; Red: Unstimulated; orange: +IL‐1*β*; blue: +IL‐6; pink: +TNF‐*α*. Results show a statistically significant increase in CB_2_ expression between controls and MS patients after stimulation with IL‐1*β*, IL‐6, and TNF‐*α*.

At the protein levels, similar trends were observed. Significance was noted in CB2 levels being higher in all cytokine‐stimulated PBMC of MS than in healthy controls (Fig. 8).

## Discussion

Our study contributes to the evidence that the interaction between the cannabinoid system and the cytokine network is bidirectional (Roche *et al*. 2008, Rubio‐Araiz *et al*. 2008) and extends this evidence to human biology. CB_1_ and CB_2_ expression levels were most significantly induced by TNF‐*α*. This occurs, at least in part, through NF‐*κ*B activation as its inhibition suppressed TNF‐*α*‐enhanced CB_1_, CB_2_ as well as TNF‐*α* gene expression itself. Activation of NF‐*κ*B could have been induced by stimulation of the TNF receptor. Upon activation, NF‐*κ*B translocates to the nucleus where it binds DNA and triggers the transcription of target genes (Ledeboer *et al*. 2005), some of which encode inflammatory proteins and may also include the cannabinoid receptor genes. It has been shown that the CB receptors are involved in TNF‐*α*‐mediated NF‐*κ*B activation (Zheng *et al*. 2008). NF‐*κ*B mediates the TNF‐*α*‐induced transcription of CB_1_ gene in neurones (Borner *et al*. 2012). Thus, cytokine‐responsive cells such as T cells, macrophages, neurones, astrocytes and microglia may enhance CB_1_ and CB_2_ expression through the NF‐*κ*B pathway. A study by Börner *et al*. (2007) provides support for this, showing that the activation of human T cells upregulates CB_1_ but not CB_2_, transcription, and that CD3/CD28‐mediated upregulation is in part NF‐*κ*B dependent through activation of CB_1_ promoter.

On the other hand, caution is invited in interpretation of our results, as SN50 may not be highly specific for NF‐*κ*B, being also a proteasome and AP‐1 inhibitor (Boothby 2001). Of note, in a separate but similar experimental set‐up, we were unable to block the induction of CB_1_ and CB_2_ in the presence of AP‐1 inhibitor NGDA, suggesting at least that the effect of SN50 was not due to AP‐1 blockade (Jean‐Gilles L, Constantinescu CS, unpublished observations).

Activation of MAPK reflects CB receptor activity. Recently, CB_2_ was shown to be upregulated by stimulation with IL‐1*β*, TNF‐*α* or lipopolysaccharide in synovial fibroblasts of patients with rheumatoid arthritis, and the selective CB_2_ agonist HU‐308 inhibited IL‐1*β*‐induced production of IL‐6 and IL‐1*β*‐induced activation of Erk 1/2 and p38 MAPK (Gui *et al*. 2014). In our study, the upregulation of CB_1_ and CB_2_ mRNA and protein was paralleled by activation of MAPK upon exposure to pro‐inflammatory cytokines. This confirms that the upregulation of CB receptors in immune cells has functional consequences. However, the similarity of MAPK activation by cannabinoid receptor activation and by inflammatory cytokines in PBMC does not necessarily imply that these phenomena are causally linked.

Our finding of upregulation of CB by inflammatory cytokines and in MS, together with the dysregulation of the endocannabinoid system in MS (Jean‐Gilles *et al*. 2009), raises the possibility of a therapeutic effect of cannabinoids in inflammatory diseases.

We show that cannabinoid receptors are functional in PBMC, as stimulation with HU‐210‐enhanced phosphorylation of MAPK 42/44 (also known as ERK1/2). Cannabinoids have previously been shown to activate these kinases through the activation of cannabinoid receptors (Upham *et al*. 2003). The fact that exposure of cytokine‐stimulated cells to cannabinoid receptor antagonists, SR1 and SR2 synergistically suppressed the functional response of the cannabinoid receptors to cytokines suggests that these responses were direct and specific.

The present study also demonstrates that CB_1_ and CB_2_ upregulation in the blood of patients with MS. This is in line with very recent data by Sanchez Lopez *et al*. (2015) which shows an increased expression of cannabinoid receptors in peripheral blood lymphocytes from patients with MS. Of note, in the second set of experiments comparing PBMC from healthy controls and patients with MS, the upregulation in MS was less prominent. The difference from baseline levels in Figure 7b may represent difference between whole blood and PBMC expression. This may also reflect the use of frozen cells (3–5 months) or the blunting of the overall expression due to the 58% of patients being on disease‐modifying treatments, as interferon treatment reduces CB levels (Sanchez Lopez *et al*. 2015). In our study, there is baseline variation in the levels of expression of these receptors, and some discrepancies between results at the mRNA and protein level; between results of whole blood testing vs. PBMC; and between results using higher vs. lower cytokine concentrations. Regulation of expression at the protein and mRNA level can differ, in particular for cytokines, through post‐translational or mRNA stability changes (e.g. Kazanecki *et al*. 2007). Nevertheless, the tendency towards an increase in CB expression by cytokines and an increase in MS compared to controls was reasonably consistent at the mRNA, protein and signalling level. The responses of immune cells of patients with MS to inflammatory cytokines, while, in some instances, more vigorous than those of controls, tend to be similar. This was also observed in immune cell subsets of a separate set of patients with MS vs. controls (*n* = 5 and 3, respectively) (data not shown); however, the small sample size precludes definitive conclusions.

Upregulation of CB in MS is associated with a simultaneous upregulation of pro‐inflammatory cytokine expression in immune cells as previously demonstrated in MS mouse models (Maresz *et al*. 2005, Loría *et al*. 2008). Our current findings are consistent with previous reports demonstrating that pro‐inflammatory cytokines are upregulated in patients with MS (Kahl *et al*. 2002, Filion *et al*. 2003).

We recognize that the association of the upregulation of CB_1_ and CB_2_ to that of cytokines in MS does not imply causation, in particular as concentration of cytokines likely to be found in body fluids in MS was less potent than the doses used *in vitro* on control cells in the first part of the study. The increased expression of CB_1_ and CB_2_ in MS may be in part mediated by individual pro‐inflammatory cytokines or their combined effect. In addition, other factors in the MS blood or inflammatory/neurodegenerative micro‐environment may also contribute to CB upregulation, as a more general damage signal. Endocannabinoids themselves can upregulate their own receptors. We have previously demonstrated upregulation of endocannabinoid levels in blood (Jean‐Gilles *et al*. 2009), which can contribute to CB_1_ and CB_2_ upregulation.

This study is among the first thorough investigations of cannabinoid receptor regulation by prototypical, antigen‐presenting cell‐derived pro‐inflammatory cytokines in humans and of their expression in MS blood. Further investigations are required to better understand the mechanisms entailed in CB_1_ and CB_2_ receptor regulation by cytokines, including studies to determine whether there is a direct transcriptional effect of these cytokines on the CB receptor genes as shown for transcriptional control of the CB_1_ gene by IL‐4 (Borner *et al*. 2008). Further investigations in MS patients, in different phases of MS with different levels of disease activity and under different treatments, will also be important. Very recently, it was shown that untreated patients with MS have increased gene expression levels of cannabinoid receptors in different types of immune cells, and those levels decrease significantly over the course of 1 year of interferon‐beta (IFN‐*β*) treatment (Sanchez Lopez *et al*. 2015). This observation is consistent with our findings, given the suppressive effect of IFN‐*β* on proinflammatory cytokines.

There may be potential limitations of our study. The specificity of CB_1_ and CB_2_ antibodies used in the first part of the study has been subject to some debate, in part explaining some of the discrepancies found in the literature (Grimsey *et al*. 2008). However, well‐conducted human studies have detected bands of the same size as in our Western blots, notably De Jesus *et al*. (2006), who detected pronounced bands of 60 and 37 kDa ascribed to CB_1_ (the latter potentially representing a result of deglycosylation or proteolysis of the latter); these bands were confirmed in other studies, for example Xu *et al*. (2005). For CB_2_, a 60 kDa band has also been confirmed, for example by Nunez *et al*. (2004). In addition, in preliminary studies, we showed significant suppression of both CB_1_ and CB_2_ positivity by an average of 74% after blocking with recombinant CB_1_ and CB_2_ (data not shown). This indicates that the antibodies used here were specific. It must be noted that densitometry on Western blots performed with HRP reaction, while not fully quantitative, allows for comparisons within experiments.

The lower cytokine concentrations were less potent than the high concentrations used initially. We need to take into account the fact that the *in vivo* inflammatory environment is much more complex and combination of lower concentrations may have similar effects to individual higher concentrations *in vitro*. For example, cytokines are known to influence the expression of one another and a multitude of other immune factors could further modulate the expression levels of cytokines or cannabinoid receptors. This is in part the reason we included experiments on whole blood in this study, to reproduce better the *in vivo* conditions and to minimize alterations that may result from cell separation. Indeed, there were differences between whole blood and PBMC results, which can be explained by the different micro‐environment. Thus, caveats of translating *in vitro* results to the complex *in vivo* context must be taken into consideration.

There may be multiple functional implications of the CB regulation by inflammatory cytokines in particular TNF‐*α*, but also IL‐6 and IL‐1. As well as cannabinoids themselves, these cytokines have both pro‐ and anti‐inflammatory properties, depending on a variety of factors including concentration, micro‐environment, timing of appearance and clearance. TNF‐*α* has both neuroprotective and neurodegenerative effects. Such dual effects are also thought to exist in CB signalling. Interesting data show a high level of coregulation between TNF‐*α* and cannabinoid receptors in the CNS, in the process of neural stem cell migration. TNF‐*α* provides a crucial signal for stem cell migration, and this signal appears to be dependent on CB_1_/CB_2_ signalling (Rubio‐Araiz *et al*. 2008). Our data provide a plausible mechanistic explanation for this important biological phenomenon, whereby TNF‐*α* induces the required CB_1_ and CB_2_ expression.

Previous studies have shown analgesic effects of CB agonists in neuropathic, but not normal, rats (Sagar *et al*. 2005). Upregulated CB_1_ expression in these animals contributes to increased analgesic efficacy of cannabinoids (Siegling *et al*. 2001). Moreover, CB_2_‐mediated control of neuropathic pain in mice is IFN‐*γ* dependent (Racz *et al*. 2008). Also, it has been shown that expression of endocannabinoid ligands is increased in a viral model of MS and inflammatory lesions of patients with MS (Eljaschewitsch *et al*. 2006, Loría *et al*. 2008) and in plasma of patients with different types of MS (Jean‐Gilles *et al*. 2009). Such studies may help to elucidate cannabinoid mechanisms involved in controlling MS symptoms such as spasticity, overactive bladder and pain. In addition, polymorphisms in the CB genes have been associated both with MS subtypes and with surface CB expression (Woolmore *et al*. 2008, Ramil *et al*. 2010, Rossi *et al*. 2013). This may explain the heterogeneity at baseline and upon stimulation in our study. Future studies may determine which patients with MS are most likely to show benefit from modulation of the cannabinoid system. If the regulation of cannabinoid receptors in CNS cells and immune cells is similar, our results could contribute to the future studies of cannabinoid neuroprotective mechanisms and possibly future treatment of inflammatory and neurodegenerative diseases.

## Conflict of interest

The authors report no conflict of interests.

This study was supported in part by the MS Society of Great Britain and Northern Ireland and by the University of Nottingham Neuroscience at Nottingham, N@N). LJE was supported by a Patrick Berthoud Fellowship. RT was supported by a Visiting Fellowship to University of Nottingham from the European Neurological Society.
